# Feasibility of Chest Wall and Diaphragm Proprioceptive Neuromuscular Facilitation (PNF) Techniques in Mechanically Ventilated Patients

**DOI:** 10.3390/ijerph19020960

**Published:** 2022-01-15

**Authors:** Tomasz Zwoliński, Magdalena Wujtewicz, Jolanta Szamotulska, Tomasz Sinoracki, Piotr Wąż, Rita Hansdorfer-Korzon, Andrzej Basiński, Rik Gosselink

**Affiliations:** 1 Department of Physical Therapy, Faculty of Health Sciences, Medical University of Gdańsk, 80-210 Gdansk, Poland; jolanta.szamotulska@gumed.edu.pl (J.S.); rita.hansdorfer-korzon@gumed.edu.pl (R.H.-K.); 2 Physiotherapy Department, University Clinical Center, 80-219 Gdansk, Poland; tsinoracki@gmail.com; 3 Department of Anaesthesiology and Intensive Therapy, Faculty of Medicine, Medical University of Gdańsk, 80-210 Gdansk, Poland; magdalena.wujtewicz@gumed.edu.pl; 4 Department of Nuclear Medicine, Faculty of Health Sciences, Medical University of Gdańsk, 80-210 Gdansk, Poland; piotr.waz@gumed.edu.pl; 5 Department of Nursing and Medical Rescue, Institute of Health Sciences, Pomeranian University in Słupsk, 76-200 Slupsk, Poland; andrzej.basinski@wp.pl; 6 Department Rehabilitation Sciences, Faculty Movement and Rehabilitation Sciences, University Hospitals Leuven, 3000 Leuven, Belgium; rik.gosselink@kuleuven.be

**Keywords:** proprioceptive neuromuscular facilitation (PNF), intensive care units (ICUs), mechanical ventilation (MV), physical therapy techniques

## Abstract

Physical therapy is part of the treatment for patients admitted to ICU. Proprioceptive neuromuscular facilitation (PNF) is one of the physiotherapy concepts including manual techniques and verbal stimulation. The purpose of this paper is to examine the feasibility of PNF techniques in mechanically ventilated (MV) ICU patients. Another aim is to verify whether the technique using resistance during the patient’s inhalation will have a different effect than the technique used to teaching the correct breathing patterns. Methods: Patients admitted to tertiary ICU were enrolled in this study, randomly divided into two groups, and received four 90-second manual breathing stimulations each. The following vital signs were assessed: HR, SBP, DBP, and SpO_2_. Results: 61 MV ICU adult patients (mean age 67.8; 25 female and 36 male) were enrolled in this study. No significant differences in HR, SBP, and DBP were observed both for two techniques measured separately and between them. Statistically significant differences were noticed analysing SpO_2_ in the rhythmic initiation technique (RIT) group (*p*-value = 0.013). Conclusions: Short-term PNF interventions did not influence clinically relevant vital parameters among MV patients and seem to be feasible in this group of ICU patients.

## 1. Introduction

Physical therapy is an inherent part of medical procedures applied in intensive care units (ICU) [1]. Several physiotherapy procedures such as respiratory muscle training, treatment for airway clearance, body positioning, mobilization, and exercise are employed to prevent and attenuate critical illness complications in ICU patients [2,3,4,5]. Professional, expensive, and single-use medical devices are required to perform the vast majority of physical therapy interventions in ICUs. The physiotherapy techniques used in the critically ICU patients should not cause any harm and should be easy to perform in those immobilized patients.

Proprioceptive neuromuscular facilitation (PNF) is a widely used physical therapy approach [6], which is most commonly used to enhance mobility, motor learning, and motor control in a wide variety of patient populations [7,8,9,10,11,12]. This treatment has also been shown to be beneficial in a healthy population to increase expiratory reserve volume (ERV) and vital capacity (VC) [13], forced vital capacity (FVC), and peak expiratory flow (PEF) [14]. Among chronic obstructive pulmonary disease (COPD) patients, PNF stretching techniques combined with aerobic training improved the outcomes of FVC, forced expiratory volume in the first second (FEV1), inspiratory capacity (IC), inspiratory reserve volume (IRV), 6-min walk test (6MWT), COPD Assessment Test (CAT), dyspnoea Visual Analogue Scale (VAS), and range of motion (ROM) [15]. PNF stimulation was verified among stroke patients and has shown to decrease tightness of accessory expiratory muscles [16], to increase the FEV1/FVC ratio [17], to improve the 6MWT, but had no influence on VC, FVC, FEV1, and 10-minute timed walking test [18]. Moreover, clinical studies have shown that manual chest and diaphragm stimulations similar in methodology to PNF techniques can increase transcutaneous oxygen saturation (SpO_2_) and thoraco–abdominal motion (TAM) in myotonic dystrophy [19], and did not disturb haemodynamic and respiratory parameters [19,20,21]. However, there is a need to evaluate the clinical utility of PNF stimulation in patients admitted to the intensive care unit (ICU). Therefore, the purpose of this study was to explore whether PNF manual interventions influence vital signs in patients on MV and are feasible and safe while applying physical therapy in this group of patients.

## 2. Materials and Methods

### 2.1. Participants

**Figure 1:**
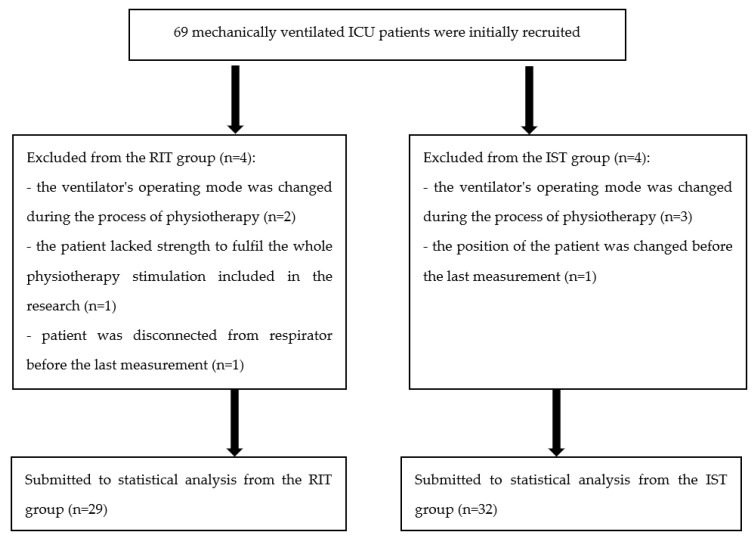
Flow chart of the study. RIT = rhythmic initiation technique, IST = initial stretch technique.

This study was a randomized clinical trial. Initially, 69 subjects were enrolled. The patients considered suitable for this study were randomly assigned to either rhythmic initiation technique (RIT) or initial stretch technique (IST) group. The flow chart of the study is shown in Figure 1.

### 2.2. Inclusion Criteria

The inclusion criteria were as follows: (1) adults of both sexes, (2) conscious and cooperative, (3) mechanically ventilated in assisted ventilation mode, (4) haemodynamically stable.

### 2.3. Exclusion Criteria

The exclusion criteria were as follows: (1) fever above 38.3 °C [22], (2) haemodynamically unstable patients, (3) thoracic or abdominal surgery precluding the use of PNF exercises, (4) rib fractures.

### 2.4. Study Design

The RIT and IST groups received one session by a physiotherapist specialized in PNF techniques (TZ, TS) and the treatment consisted of four 90-second manual stimulations each (upper ribs, lower ribs, sternum, and diaphragm techniques). Vital signs were assessed before and after the session.

The RIT was performed to teach coordination of motion and to establish the correct breathing pattern [23,24]. The IST was applied to reinforce the strength of inspiratory muscles. Its main aim is to facilitate the initiation of motion [23,25].

During the treatment the ventilator operating mode (pressure support ventilation) did not change. Moreover, from the beginning of the rehabilitation until the last measurement, the patient’s body position was unchanged. After data collecting, general physiotherapy session was allowed in both groups, adjusted to the health condition of the patient.

The research was carried out from 5th November 2016 until 29th October 2018 and was conducted in the Anaesthesiology and Intensive Therapy Unit of the University Clinic Centre in Gdansk, Poland. A consent NKBBN/444-179/2016 for conducting this research was given by the Independent Bioethics Committee for Scientific Research at the Medical University of Gdansk, Poland.

### 2.5. Protocol

PNF techniques included one session of physiotherapy including four 90-second manual stimulations each (upper ribs, lower ribs, sternum, and diaphragm). After every stimulation the patient rested for one minute. Conscious and cooperative patients were instructed by the physiotherapist (PT) to follow commands.

The first group was treated with the RIT derived from the PNF concept [23]. This technique facilitates the correct movement pattern, improves coordination and movement awareness of the chest wall. The patient was led from a passive to active movement with the use of a directional resistance [24]. The RIT was applied in four manual positions of the therapist’s hands: The upper and lower chest wall, the sternum and below the rib cage, so that the patient could learn the correct breathing pattern. Verbal commands were also used by the PT to reinforce the manual stimulation.

**Figure 2:**
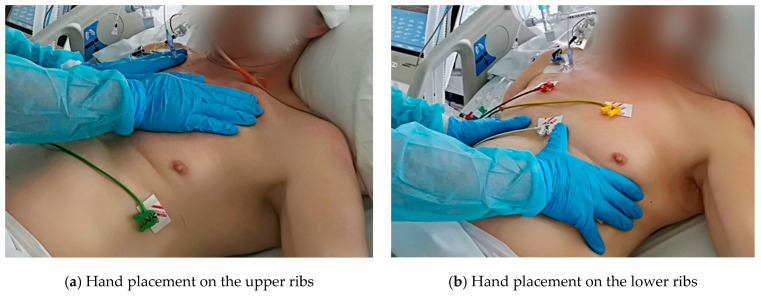

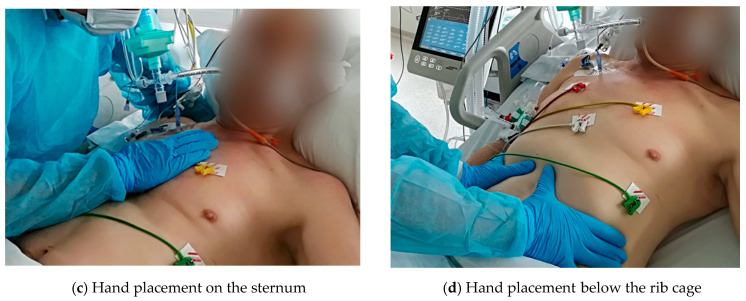
(**a**–**d**) Hand placement during breathing stimulation for both the RIT and IST. RIT = rhythmic initiation technique, IST = initial stretch technique.

At the beginning of the session, the PT kept his/her hands on the upper part of the chest wall, just below the clavicles, supporting the patient’s exhalation phase by guiding the movement. Subsequently, a slight posterior caudal pressure was applied to the chest wall using a verbal cue “exhale”. While inhaling, the physiotherapist’s hands exerted a slight direct resistance against the expanding upper ribs, giving the patient a perception of the movement and helping to perform it. At the same time, the patient was verbally encouraged to take a deep breath. (Figure 2a). The second manual application of the PT was on the lower lateral regions of the chest wall. It was based on supporting the patient’s exhalation phase by a slight compression of the chest wall in the caudal medial direction, simultaneously using a verbal cue “exhale”. During the inhalation phase, the hands of the physiotherapist initially only guided the movement. Then they exerted a slight direct resistance to the lateral expansion of the lower ribs, giving the patient the perception of the movement and helping to execute it. At the same time, the patient was verbally encouraged to take a deep breath (Figure 2b). Subsequently, the PT placed his hands on the patient’s sternum, facilitating the patient’s exhalation phase by guiding and applying light pressure to the sternum in the caudo-posterior direction, simultaneously using a verbal cue “exhale”. During the inhalation phase the hands of the physiotherapist (still kept on the sternum) were initially only guiding the movement. Next the PT exerted a slight direct resistance to the expansion of the upper chest wall giving the patient the perception of the movement and helping to execute it. At the same time, the patient was verbally encouraged to take a deep breath (Figure 2c). Finally, the PT placed his/her hands on the upper abdomen, just below the costal arches and supported the patient’s exhalation phase by slight lengthening the diaphragm simultaneously in the posterior-superior direction using a verbal cue “exhale”. When stimulating the inhalation phase the hands of the physiotherapist initially only guided the inspiratory movement of the diaphragm. Then they exerted a slight posterior–superior guiding resistance against the expanding abdominal wall facilitating and stimulating the diaphragm to shorten. At the same time, the patient was verbally encouraged to take a deep breath (Figure 2d). After every stimulation mentioned above the patient rested for one minute without any physical therapy.

The second group was treated with the IST, a technique also originating from the PNF concept (named also as: repeated stretch from beginning of range or repeated initial stretch) [23]. This technique facilitates the initiation of inhalation. Performing the IST, the PT utilizes the stretch reflex response of the muscle–tendon complex in elongation to reinforce the contraction of the stretched muscles [25]. The IST was applied to help the patient to initiate the inhalation phase, increase the force developed by the inspiratory muscle, and to enhance the active range of motion of the chest wall and the diaphragm. At the final stage of exhalation, when inspiratory muscles were elongated optimally, the stretch reflex was initiated by applying a quick tap to elicit a strong and active inspiratory muscle contraction. The tap was applied in four hand placements shown in Figure 2a–d. While patient inspired, the PT performed a slight guiding resistance with his/her hands in order not to restrict the fluent movement capability of the patient, but to facilitate the range of motion. Simultaneously, the patient was verbally encouraged by the PT to take a strong and deep breath.

The IST technique involved the PT placing his hands in the same areas as for the RIT as presented in Figure 2a–d (the upper part of the chest wall, the inferior lateral part of the chest wall, the sternum, and below the rib arches). Moreover, the PT’s hands worked in the same directions during the exhalation and inhalation phases while performing both techniques.

As in the case of providing the RIT, after every stimulation using the IST, the patient was resting for 1 minute without any support from the PT.

### 2.6. Assessment

The following vital signs were assessed: heart rate (HR), systolic blood pressure (SBP), diastolic blood pressure (DBP), and percutaneous oxygen saturation (SpO_2_) in order to assess changes in the patients’ vital functions when applying the RIT and the IST from the PNF concept.

All the analysed vital signs mentioned above were measured noninvasively and collected from the bedside monitor (Philips IntelliVue MX800, Boeblingen, Germany) before, and 5 and 60 min after, the physiotherapy session.

### 2.7. Statistical Analysis

The results were generated using the R statistics language [26]. Basic statistics (mean, standard deviation, median) were calculated for quantitative variables. The Shapiro–Wilk test was used to verify the hypothesis that the quantitative data came from a normally distributed population. Comparing two samples from a population with a normal distribution, the homogeneity of their variance was checked. After determining whether the variables were related, appropriate tests were selected (*t*-test, Wilcoxon–Mann–Whitney test). The Kruskal–Wallis, Friedman, and ANOVA tests were used to compare more than two groups of data. The appropriate post-hoc tests, in the cases of statistically significant results for the above-mentioned tests, were also performed. For qualitative variables, the frequency of the analysed categories consisted the basic statistics. The assumed significance level was α = 0.05.

## 3. Results

Characteristics of the 61 patients are presented in Table 1. Patients were examined in highly specialized trauma and surgical ICU. No statistically significant differences were found between these patients (age, gender, the type of artificial airway (endotracheal tube or tracheostomy), day of the study, and ventilator settings: level of pressure support (PS), positive end-expiratory pressure (PEEP) and fraction of inspired oxygen (FiO_2_)) regardless of the technique used (RIT or IST) or the time after the technique was applied.

The medians, minimal, and maximal values of all measured vital signs, respectively, are shown in Table 2. Additionally, the *p* values were also included.

Most of patient appreciated the treatment and could fulfil it without any disturbance. Eight of them (details can be found in Figure 1) were not included in the final analysis. However, only one patient could not perform the whole session (RIT) because of the lack of strength.

There were no statistically significant differences between the RIT and the IST for all analysed vital signs in all measurement times. SpO_2_ values for the three time points (0/5/60) for patients undergoing the RIT technique were tested as shown in the Table 2. The obtained *p*-value = 0.013 allows to state that there are statistically significant differences between these values.

Having analysed the influence of RIT on SpO_2_ using the Friedman test a statistically significant difference was observed (*p*-value = 0.013). The Friedman post hoc analysis showed a statistically significant difference in the value of SpO_2_ between the measurements taken 60 and 5 min after the end of the therapy (*p*-value = 0.009). Statistically significant differences were neither found when comparing the measurements taken 60 min after physiotherapy to the measurement before therapy (*p*-value = 0.433) nor the measurements taken 5 min after physiotherapy to the measurement before therapy (*p*-value = 0.206).

In the case of the IST technique, the Friedman test was also used to investigate the differences between the SpO_2_ values at the analysed time points. The obtained *p*-value = 0.227 does not allow for the adoption of an alternative hypothesis stating the existence of differences between the analysed groups of SpO_2_ values (Table 2).

The collected HR values for the studied group of patients before the therapy, 5 and 60 min after the therapy performed with the RIT technique do not differ in terms of the Friedman test (*p*-value = 0.633).

The HR values collected for the group of patients undergoing IST therapy were tested using the ANOVA (One-way) test. The obtained *p* value = 0.924, indicates no statistically significant differences for the HR values collected before the therapy, 5 and 60 min after the therapy.

Subsequently, the ANOVA (Two-way) test was performed to analyse SBP values. The obtained *p*-value = 0.485 indicates no statistically significant differences between the SBP values due to the techniques (IST/RIT) and time (0/5/60).

Also, when assessing DBP changes due to the techniques (IST/RIT) and time (0/5/60), the obtained *p*-value = 0.717 indicates no statistically significant difference.

The observed the differences among median values of HR, SBP, DBP, and SpO_2_ when applying both techniques are shown in Table 2.

## 4. Discussion

Rhythmic initiation technique (RIT) and initial stretch technique (IST) applied in conscious mechanically ventilated patients elicited no statistically significant differences in heart rate, blood pressure, and oxygen saturation. The obtained results indicate that both techniques (IST and RIT) led to similar results and did not destabilise the clinical condition of mechanically ventilated ICU patients in relation to the analysed parameters. This indicates that interventions used in the present study can be feasible and safe when manual chest and diaphragm stimulations are applied in ICU subjects. Nitz and Burke [19] studied the effect of manual pulmonary rehabilitation using the IST and the staged basal expansion (SBE) intervention in subjects with muscular dystrophy. The first intervention included a quick stretch at the end of the exhalation, as is the case of the IST. The second stimulation (SBE) involved manual stimulation of the chest wall by the PT placing hands on the lower chest area and verbally encouraging the patient to take deep breaths, which is similar to the RIT. The researchers, in contrast to our investigation, stated that the IST increased the value of SpO_2_ more efficiently than the SBE technique. The authors concluded that the initial stretch, occurring in the IST, may affect the intercostal muscles in patients with muscular dystrophy by providing sensory stimulation to the physiological afferentation of facilitated muscles.

We suppose that patients participating in our study did not respond to the IST by increasing SpO_2_ value either because the initial stretch at the end of the exhalation was too intense stimulation for them or the influence of received medications might have compromised the inspiratory muscle response to quick stretch applied at the end of the exhalation.

Stable HR, SBP, and DBP during both PNF stimulations indicate that the techniques used were optimally adjusted to the patients’ condition. Aggressive stimulation could lead to tachycardia or induce pain and exhaustion. Nitz and Burke [19] noticed a decrease in HR after applying SBE stimulation, which is similar to SBE.

Both stimulation from the PNF concept, the IST and the RIT, improve coordination and awareness of motion [6,23]—regrettably it was not verified in this research. However, Slubska et al. [16] pointed out that manual stimulation of the chest wall and respiratory muscles using the IST concept can decrease excessively tight accessory expiratory muscles among patients after ischaemic stroke. Authors concluded that stimulating the excessively tight accessory respiratory muscles in stroke patients may improve their contractility and function, especially during deep breathing. In addition, it could prevent coordination disorders and lead to improvement of respiratory muscles function. In summary, it was concluded that applying the PNF technique in stroke patients can help them normalize breathing patterns, improve lung ventilation, and thus prevent the occurrence of hypoxia [16].

We had difficulties recruiting patients who met the inclusion criteria. Many patients in the Anaesthesiology and Intensive Therapy Unit of the University Clinic Centre in Gdańsk, Poland, are hospitalized for severe trauma and after major surgical operations. These excluded the application of the IST stimulation when the additional manual quick tap at the end of exhalation is applied. It is in this context important to emphasize that, according to Weigl et al. [27], the mortality rate in Polish ICUs is 42%. Authors stressed that in other European countries the mortality rate ranges from 6.7 to 17.8% [28,29]. They presumed that the reason for that could be the fact that many patients admitted to ICU in Poland were at very high risk of death as well as there was a low number of discharges of patients with a poor prognosis for palliative care. In the response to the abovementioned report Piechota et al. [30] conclude that, patients admitted to Polish ICUs are sicker than those admitted to ICUs in other European countries. They have higher APACHE II score: 26.0–27.1 ± 10.4 versus 15, 15.9 and 20.5 ± 8.53 in the Netherlands, Sweden, and the United Kingdom, respectively.

In the present study, no statistically significant differences were found in cardiovascular parameters. It is possible, if the amount of physical therapy had been more intense than one per day, we might have observed statistically significant changes with clinical importance. A survey of 146 ICU patients published by Castro et al. [31] showed the decrease in the length of hospital stay, in mortality, in the rate of respiratory infections, and in duration of mechanical ventilation when chest physiotherapy was performed four times per day compared to one physiotherapy session. On the other hand, applying physiotherapy one time per day reflected the actual situation in most Polish ICUs where only the tertiary level ICUs are obligated to employ PT on a half-time basis [32,33]. It can be concluded that IST and RIT can be used in ICU patients during early physical therapy treatment in mechanically ventilated ICU patients.

## 5. Limitations of the Study

Major limitations of the present study were the enrolment of 69 patients and performing the physiotherapy stimulations only once. Moreover, using arterial blood gases as well as ventilatory parameters (respiratory rate, tidal volume, and vital capacity) could help obtain more precise data on the ventilatory responses.

## 6. Conclusions

Short-term physical therapy interventions using the stimulations derived from the PNF concept did not have a clinically relevant effect on observed vital signs. Taking this into consideration, we can conclude that both stimulations are feasible and safe as a physiotherapy tool in mechanically ventilated ICU patients. Further clinical studies are needed to estimate how physical therapy interventions using PNF techniques correlate with respiratory parameters and health conditions of ICU patients.

### Practical Implications

The exerted manual mobilisation of the chest and diaphragm using the RIT and IST originating from the PNF concept could be utilized during early physical therapy of ICU subjects. Since the applied techniques normalize breathing patterns improving respiratory muscles function [16] they might support the weaning process. These require only the manual skills of the physiotherapist and could increase the variety of physical therapy interventions of ICU MV patients.

## Figures and Tables

**Table 1:** The general characteristic of the patients.

Variables	RIT Group (*n* = 29)	IST Group (*n* = 32)	*p*-Value
age: Mean value (minimum–maximum value)	69 (34–94)	66 (24–86)	0.405 ^t^
Sex—Female (%)	11 (18%)	14 (23%)	0.841 ^χ^
number of patients with endotracheal tube (%)	27 (44%)	24 (39%)	0.119 ^Y^
number of patients with tracheostomy tube (%)	2 (3%)	8 (13%)	
number of days of mechanical ventilation until the examination: median (minimum–maximum value)	5 (1–27)	8 (1–32)	0.189 ^W^
PS (cmH_2_O): Mean (standard deviation)	13 (5)	13 (5)	0.453 ^W^
PEEP (cmH_2_O): Median (minimum-maximum value)	5 (3–10)	6 (3–10)	0.285 ^W^
FiO_2_ (%): Median (minimum-maximum value)	35 (30–55)	40 (25–65)	0.698 ^t^

RIT = rhythmic initiation technique, IST = initial stretch technique, PS = pressure support, PEEP = positive end-expiratory pressure, FiO_2_ = fraction of inspired oxygen, t = Student’s *t*-test, χ = χ^2^ test of independence, Y = χ^2^ test of independence (with Yates’ continuity correction), W = Wilcoxon test.

**Table 2:** HR, SBP, DBP, and SpO_2_ before, and 5 and 60 min after, physiotherapy for the RIT and the IST.

Vital Sign Observed Median (Min–Max Values)	PNF Applied Technique	before Physiotherapy	5 min after Physiotherapy	60 min after Physiotherapy	*p*-Value
Heart Rate (HR)	RIT	88 (43–147)	81 (42–157)	85 (50–122)	0.633 ^F^
	IST	86 (53–112)	87 (54–145)	84 (49–141)	0.924 ^O^
Systolic Blood Pressure (SBP)	RIT	126 (98–168)	126 (85–188)	124 (77–156)	0.485 ^T^
	IST	132 (94–171)	132 (84–176)	129 (101–197)	
Diastolic Blood Pressure (DBP)	RIT	67(36–96)	66 (45–103)	65 (35–101)	0.717 ^T^
	IST	68 (39–104)	71 (45–97)	69 (49–101)	
Oxygen Saturation (SpO_2_)	RIT	97 (87–100)	97 (89–100)	98 (91–100)	0.013 ^F^
	IST	97 (87–100)	96 (88–100)	97 (88–100)	0.227 ^F^

RIT = rhythmic initiation technique, IST = initial stretch technique, F = Friedman test, O = One-way ANOVA test, T = Two-way ANOVA test.

## Data Availability

The authors declare that the data from this study are available from the author P.W. (piotr.waz@gumed.edu.pl) upon request.
